# Experimental Microbial Alteration and Fe Mobilization From Basaltic Rocks of the ICDP HSDP2 Drill Core, Hilo, Hawaii

**DOI:** 10.3389/fmicb.2018.01252

**Published:** 2018-06-14

**Authors:** Marius Stranghoener, Axel Schippers, Stefan Dultz, Harald Behrens

**Affiliations:** ^1^Institute of Mineralogy, Leibniz Universität Hannover, Hanover, Germany; ^2^Geomicrobiology, Federal Institute for Geosciences and Natural Resources, Hanover, Germany; ^3^Institute of Soil Science, Leibniz Universität Hannover, Hanover, Germany

**Keywords:** Fe mobilization, volcanic islands, ICDP, microbe-basalt interaction, bio-mediated alteration, biofilm, *Burkholderia*, Fe redox state

## Abstract

The interaction of a single bacterial species (*Burkholderia fungorum*) with basaltic rocks from the ICDP HSDP2 drill core and synthetic basaltic glasses was investigated in batch laboratory experiments to better understand the role of microbial activity on rock alteration and Fe mobilization. Incubation experiments were performed with drill core basaltic rock samples to investigate differences in the solution chemistry during biotic and abiotic alteration. Additionally, colonization experiments with synthetic basaltic glasses of different Fe redox states and residual stresses were performed to evaluate their influence on microbial activity and surface attachment of cells. In biotic incubation experiments bacterial growth was observed and the release of Fe and other major elements from drill core basaltic rocks to solution exceeded that of abiotic controls only when the rock sample assay was nutrient depleted. The concentration of dissolved major elements in solution in biotic colonization experiments with synthetic basaltic glasses increased with increasing residual stress and Fe(II) content. Furthermore, the concentration of dissolved Fe and Al increased similarly in biotic colonization experiments indicating that their dissolution might be triggered by microbial activity. Surface morphology imaging by SEM revealed that cells on basaltic rocks in incubation experiments were most abundant on the glass and surfaces with high roughness and almost absent on minerals. In colonization experiments, basaltic glasses with residual stress and high Fe(II) content were intensely covered with a cellular biofilm. In contrast, glasses with high Fe(III) content and no residual stress were sparsely colonized. We therefore conclude that structurally bound Fe is most probably used by *B. fungorum* as a nutrient. Furthermore, we assume that microbial activity overall increased rock dissolution as soon as the environment becomes nutrient depleted. Our results show that besides compositional effects, other factors such as redox state and residual stress can control microbial alteration of basaltic glasses.

## Introduction

Weathering of volcanic rocks is a key factor for the transport and geochemical cycling of elements between lithosphere and hydrosphere. The oceanic crust is composed mainly of glassy or massive basaltic lava flows that cover 60 % of the earth’s surface (Nielsen and Fisk, 2010). Volcanic rocks often show high contents of glass when erupted in contact with water due to rapid cooling. The corresponding rocks can be composed of small glass fragments (hyaloclastites) or have a glassy rim and a partially crystallized interior (pillow basalts). As a relic from rapid cooling, such glasses show residual stress weakening their structure and making them more vulnerable to alteration (Staudigel and Hart, 1983). Environmental influences such as water, temperature and atmospheric gases induce weathering and alter the chemical composition by the dissolution of primary and formation of secondary mineral phases. Basaltic rocks are in general less resistant to weathering when compared with other volcanic (e.g., rhyolites) or intrusive rocks (e.g., granites) due to their low silica and high glass content, respectively (Wolff-Boenisch et al., 2004). Young volcanic islands comprising relatively fresh and chemically reactive basaltic rocks are a potential source for increased Fe supply to ocean surface waters. Dissolved Fe in the Pacific surface waters was found to increase near the Hawaiian Islands from 0.1 nM to 0.6 nM with a maximum of 1.56 nM close to the Kauai Coast (Brown et al., 2005).

The mechanisms of chemical glass and rock alteration have been extensively studied in the past (Furnes, 1975; Seyfried and Bischoff, 1979; Oelkers, 2001; Gislason and Oelkers, 2003; Franke, 2009; Gudbrandsson et al., 2011; Dultz et al., 2016). However, we are still beginning to understand the contribution of microorganisms to these processes. Microbial activity in natural basaltic rocks is evident from alteration textures, major element chemistry and geochemical tracers at temperatures up to 100°C and as deep as 380 meter below seafloor (Thorseth et al., 1992; Torsvik et al., 1998; Furnes et al., 1999; Furnes and Staudigel, 1999; Alt and Mata, 2000; Walton, 2008). Biologically mediated alteration of volcanic glasses was also observed in samples from ICDP/IODP drilling sites making a major contribution of up to 75% to total alteration (Furnes and Staudigel, 1999; Staudigel et al., 2008). Hyaloclastites from HSDP (Hawaii Scientific Drilling Project) drill hole indicate microbial activity in rocks to depth of 1.9 km and possibly deeper (Fisk et al., 2003). Nevertheless, there are only a few studies that have investigated dissolution mechanisms of basaltic glasses under the action of microorganisms. Bacteria collected near deep sea hydrothermal vents are capable of inducing the release of Fe and Mn from basaltic rocks and contribute to the geochemical cycling of these elements (Daughney et al., 2004). Bailey et al. (2009) have shown in laboratory experiments that microbes are able to obtain beneficial nutrients from basaltic glasses in a nutrient- and energy limiting environment. Furthermore, basaltic glasses are a potential habitat for Fe(II)-oxidizing microorganisms using Fe(II) in basaltic glasses as an energy source (Henri et al., 2016; Sudek et al., 2017). Microbial oxidation of Fe(II) by different strains of Fe-oxidizing bacteria (FeOB) was found to enhance the dissolution of basaltic rocks by six to eight times over abiotic rates (Edwards et al., 2004). Wu et al. (2007) also observed elevated elemental release rates during microbial alteration of basaltic rocks relative to inorganic conditions. In contrast, other studies reported small to negligible effects of microbial activity on basaltic glass dissolution rates (Stockmann et al., 2012). Such discrepancies are believed to result from different experimental approaches and initial nutritional status of the bacteria (Perez et al., 2016).

Fe is a micronutrient for most organisms and as such essential for a variety of enzymatic processes (Rogers and Bennett, 2004). It is found in nature in its two oxidations states as Fe(II) and Fe(III). The solubility of Fe in natural fresh- and seawaters and therefore its bioavailability depends on several parameters such as pH, oxygen availability, redox potential, and the presence of organic ligands. At neutral pH and in oxygen rich environments, Fe is mostly present as insoluble Fe(III) forming Fe oxy(hydroxides). However, microorganisms have developed strategies to overcome this problem by creating reactive microenvironments at the mineral or glass surface. The most important are (i) complexation or oxidation/reduction of ions changing the equilibrium of the system (Stockmann et al., 2012; Navarrete et al., 2013) and (ii) bacterial acid production and proton consumption that change the solution pH and in this way enhancing dissolution and release of limiting nutrients (e.g., Fe, P) (Wu et al., 2007). The majority (>99%) of microorganisms in aqueous and soil environments are attached to surfaces and alteration studies have shown that surface attached microorganisms are by far more effective in dissolution of nutritional elements than unattached microorganisms (Ahmed and Holmström, 2015). Furthermore, the abundance of microorganisms is positively correlated with the age and alteration state of basaltic rocks (Santelli et al., 2009).

It is known from field studies that microbial colonization and alteration is restricted to minerals and glasses containing beneficial elements (e.g., Fe, P) (Rogers and Bennett, 2004). A loss of Fe(II) and Mn(II) and oxidation of residual Fe in subseafloor basaltic glasses was evidenced close to microbial alteration features (Knowles et al., 2013). In addition, Templeton et al. (2005) found Mn(II) oxidizing bacteria on pillow basalts from Loihi Seamount, which supports a biological catalysis of Mn(II) oxidation during basalt weathering. The microbial release of Fe and Mn from rocks into solution is also assumed to be relevant for the formation of Mn-nodules on deep sea sediments (Blöthe et al., 2015).

In this study, batch experiments were performed with a single bacterial species (*Burkholderia fungorum*) under laboratory conditions to investigate the potential role of microorganisms in the alteration and Fe mobilization from different natural basaltic rocks from the ICDP HSDP2 drill core. In addition, synthetic basaltic glasses were used as an analogue to specifically study the importance of the Fe redox state and residual stress on microbial activity. Biotic and abiotic control experiments were performed in a nutrient depleted medium and changes in solution chemistry were continuously monitored. Surface morphological features were analyzed after 41/42 days by environmental scanning microscopy and laser microscopy. We tested the hypothesis that microbial activity results in increased Fe mobilization and element release relative to abiotic conditions when the microorganisms are forced to actively scavenge nutrients from the basaltic rocks.

## Materials and Methods

### Site Description

**FIGURE 1:**
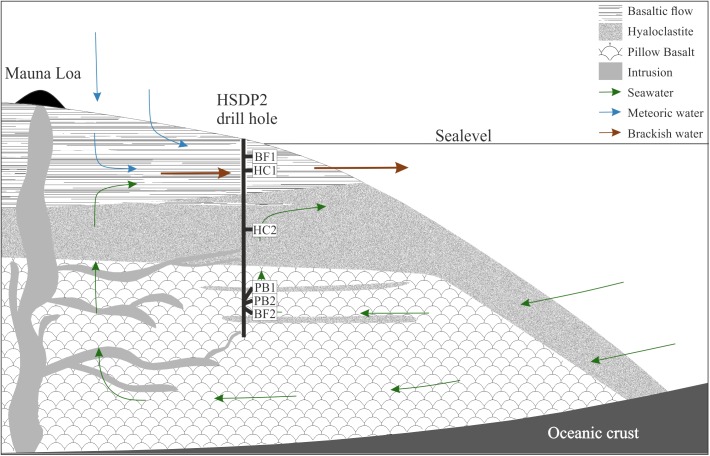
Schematic overview of the drilling site. Shown are the main lithologies found in the drill core and a simplified illustration for the hydrology of the island of Hawaii. The relative sample depth of the natural basaltic rocks used in incubation experiments are shown as well (modified after Garcia et al., 2007).

The Hawaiian emperor chain comprises five main islands and more than 100 sea mounts. All of them are of volcanic origin, formed by mantle plume activity. The island of Hawaii is the youngest, largest, and the only one with active volcanism. The hydrology of the island is complex as it is not solely composed of a surface near freshwater aquifer overlaying seawater derived saline fluids but also by alternating layers of fresh- and seawater in greater depth (Thomas and Conrad, 1996). Exchange in the circulating seawater system is fast enough to keep temperatures of ∼9°C to depth of 1600 mbsl (mbsl = meter below sea level), which may be responsible for the relatively unaltered rocks observed in the drill core (Walton and Schiffman, 2003; Stolper et al., 2009). Below this depth, the temperature gradient slowly increases with 19°C/km to 45°C at depth of ∼3 km. The majority of weathering products on the island of Hawaii are transported via submarine groundwater discharge to the ocean as a consequence of highly permeable volcanic rocks (Schopka and Derry, 2012). This is shown in **Figure 1** where cold seawater penetrates the highly porous rocks and flows landwards. The seawater then rises towards the freshwater lens, mixes, and discharges as brackish water to the ocean surface waters.

**Figure 1** also shows a schematic overview of the drilled lithologies as well as the hydrological conditions and the relative sample locations in the ICDP HSDP2 drill core (Garcia et al., 2007). The core was drilled to a total depth of 3098 mbsl including subaerial (= erupted above sea level; from surface to 1079 mbsl) and submarine (= erupted below sealevel; from 1079 mbsl to total depth) volcanic rocks.

The rock types included hyaloclastites, pillow basalts, basaltic flows, and to a lesser extent, intrusive rocks. Hyaloclastites are classified as a breccia composed mainly of glass with olivine phenocrysts and minor amounts of plagioclase and pyroxene (Walton et al., 2005). Pillow lavas are characterized by glassy margins originated from submarine quenching and lower olivine contents compared to basaltic flows. Intrusive rocks occurred only at depth >1883 mbsl and were petrographically and geochemically distinct from other core rocks (Rhodes and Vollinger, 2004; Stolper et al., 2004). Alteration of core rocks was in general less pronounced and mostly restricted to fractures, vesicles, and olivines, leaving major portions of the rocks relatively fresh (Garcia et al., 2007).

### HSDP2 Core Rocks

**FIGURE 2:**
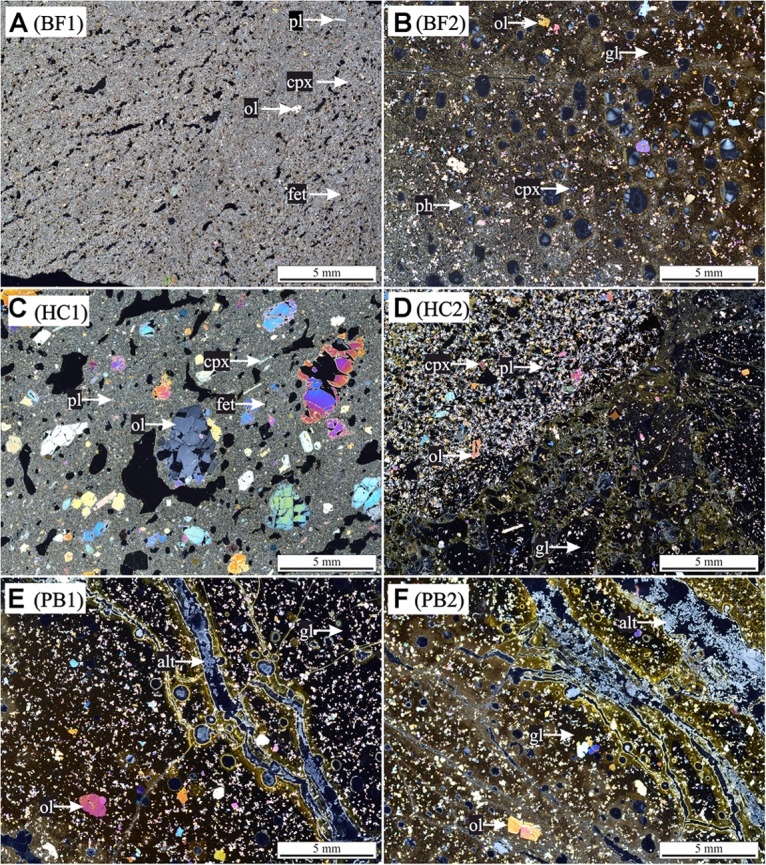
Thin section photographs with cross-polarized light of HSDP2 drill core samples used for incubation experiments. **(A)** basaltic flow (BF1) with cryptocrystalline matrix [pl (plagioclase) + ol (olivine) + cpx (clinopyroxene) + fet (Fe-Ti oxides)]. **(B)** Basaltic flow (BF2) with gl (glass matrix) + ol + cpx and vesicles filled with ph (phillipsite). **(C)** Hyaloclastite (HC1) with cryptocrystalline matrix (pl + ol + cpx + fet) + ol + cpx + pl phenocrysts. **(D)** Hyaloclastite (HC2) with gl + ol + cpx + pl phenocrysts. **(E)** Pillow basalt (PB1) with gl + ol phenocrysts + alt (alteration products) in veins. **(F)** Pillow basalt (PB2) with gl + ol phenocrysts + alt in veins. The glass in all samples is partially altered to palagonite (brownish colored).

Samples of the ICDP HSDP2 drill core were achieved from the repository at the American Museum of Natural History in New York. Six different basaltic rock samples of various sampling depths were chosen for the experiments, namely two basaltic flows (BF1: 604 mbsl; BF2: 2399 mbsl), two hyaloclastites (HC1: 732 mbsl; HC2: 1904 mbsl) and two pillow basalts (PB1: 2377 mbsl; PB2: 2385 mbsl). The samples were analyzed by polarization microscopy for their mineral content and texture (**Figure 2**). Considering their petrography, all samples contained olivine- ((Mg,Fe(II))_2_[SiO_4_]) and clinopyroxene ((Mg,Fe(II),Mn(II))(Mg,Fe(II),Mn(II),Ca)[Si_2_O_6_]) phenocrysts in different proportions (5–20 vol% olivine; 5–15 vol% clinopyroxene). Plagioclase ((Ca,Al)_2_[Si_2_O_8_] and/or (Na,Al)_2_[Si_3_O_8_]) phenocrysts were present in samples BF1, HC1, and HC2 (5–20 vol%). Secondary minerals such as phillipsite ((K,Na,Ca_0.5_)_6_[Al_6_Si_10_O_32_]^∗^12H_2_O) were observed in both pillow basalts PB1 and PB2 (veins) and sample BF2 (vesicles). In most samples, the matrix was composed of glass (60–90 vol%) with some showing small quench crystals of plagioclase (BF2, PB1, PB2). The glass was partially altered to palagonite along fractures. Samples BF1 and HC1 were the only samples with a cryptocrystalline matrix that was composed of different proportions of plagioclase, olivines, clinopyroxenes, and Fe-Ti-oxides and only minor interstitial glass.

### Preparation of Core Samples

We used coarse rock fragments with relatively small contributions of fresh surfaces. Hence, core samples were gently crushed and sieved to separate the 1–2 mm and 0.2–1 mm fractions to minimize problems of element release from highly reactive fresh surfaces (due to sawing and breaking of samples). Fine particles were removed by washing with ultrapure H_2_O in an ultrasonic bath (Sonorex RK1028, Bandelin) for 30 s. The suspension was discarded and the procedure repeated until no fine particles were observed. 350 mg of the 1–2 mm and 150 mg of the 0.2–1 mm fraction ( = 0.5 g in total) were mixed and used for each experimental assay. The specific surface area (SSA) of core samples was determined using a multi-point N_2_ adsorption BET method (Nova 4000e). SSAs of samples are 3.05 m^2^/g (BF1), 28.89 m^2^/g (BF2), 3.07 m^2^/g (HC1), 33.73 m^2^/g HC2, 20.23 m^2^/g (PB1) and 43.78 m^2^/g (PB2). Furthermore the amount of secondary amorphous and crystalline Fe, Al, and Si bearing phases was quantified using oxalate (17.5 g/L C_2_H_2_O_4_ + 28.4 g/L Na_2_C_2_O_4_) and dithionite-citrate-bicarbonate (70.4 g/L Na_3_C_6_H_5_O_7_ + 16.8 g/L NaHCO_3_ + 0.5g Na_2_S_2_O_4_) sequential extraction methods (for detailed description see Mehra and Jackson, 1960 and Schwertmann, 1964). These methods provided estimates on the proportions of different alteration phases and allowed a characterization of core samples with respect to their degree of alteration.

### Preparation of Synthetic Basaltic Glasses

Natural rocks are heterogeneous concerning their mineral assemblage and degree of alteration. During submarine volcanic eruptions the basaltic melt is rapidly quenched (cooled) by its contact with seawater. The huge temperature differences between the surface (in contact with water) and the interior produces compressive stresses (interior) and tensile stresses (surface) that favor fracturing (Narayanaswamy and Gardon, 1969). The residual stress can be released by heating the glass just below its glass transition temperature and then slowly (<1 K/min) cooling down to room temperature (annealing). Synthetic basaltic glasses with homogenous composition and known properties were used to investigate the influence of the Fe redox state and residual stress on microbial alteration and cell attachment. The synthetic glass was prepared from high purity powdered oxides and carbonates (SiO_2_, Al_2_O_3_, Fe_2_O_3_, CaCO_3_, MgCO_3_, Na_2_CO_3_, TiO_2_, K_2_CO_3_, (NH_4_)H_2_PO_4_) and had the same major element composition than the natural glasses from HSDP2 drill core (**Table 1**). Melting was done in a platinum crucible at 1600°C for 2 h. The melt was quenched on a brass plate, crushed using a steel mortar and re-melted for an additional hour at 1600°C. A portion of the melt was then poured on a hot (550°C) steel plate, put in a chamber furnace at 550°C and slowly (<1 K/min) cooled down to room temperature to remove internal stresses from the glass (sample BAS1). This glass will be considered as “stress-free” (annealed). The rest of the melt was quenched on a brass plate (sample BAS2). These two glasses were melted in air (log(fO_2_/bar) = -0.37) and were considered as oxidized glasses. A portion of the quenched glass was crushed to a fine powder (<64 μm) and put in a horizontal reduction furnace to achieve a certain Fe redox state. Here the powder was reduced below its glass transition temperature (T_g_) at 550°C for 24 h using Ar/H_2_ gas mixture (log(fO_2_/bar) = -21.53). The reduced glass powder was then melted in a corundum crucible with a graphite lid on top at 1600°C for 5 min and small melt droplets were poured out and quenched on a brass plate (sample BAS3). Samples BAS2 and BAS3 were glasses with high residual stress similar to those in hyaloclastites and glassy margins of pillow basalts. The residual stress was not measured but the synthetic glasses could be seen as “endmembers” (no residual stress vs. high residual stress). The Fe redox state of the samples was determined by a wet chemistry method of Wilson (1960) modified by Schuessler et al. (2008). Fe^2+^/Fe_tot_ ratios were 0.36 (BAS1) and 0.37 (BAS2) for glasses melted in air and 0.86 (BAS3) for the glass melted under reduced conditions (Ar/H_2_).

**Table 1:** Composition of the synthetic glass BAS and its natural analog.

Oxide	BAS [wt%]	Garcia (1996)^c^ [wt%]
SiO_2_	54.01 ± 0.48^b^	52.07 ± 0.42^d^
TiO_2_	2.82 ± 0.04	2.81 ± 0.42
Al_2_O_3_	12.70 ± 0.19	13.05 ± 0.35
FeO_tot_^a^	11.74 ± 0.44	11.80 ± 0.30
MnO	0.20 ± 0.09	0.18 ± 0.00
MgO	6.11 ± 0.12	6.26 ± 0.14
CaO	10.85 ± 0.18	10.51 ± 0.27
Na_2_O	2.13 ± 0.08	2.23 ± 0.12
K_2_O	0.41 ± 0.02	0.47 ± 0.05
P_2_O_5_	0.27 ± 0.09	0.30 ± 0.02
Total	101.26 ± 0.91	99.70 ± 0.12


*^*a*^All Fe expressed as FeO; ^*b*^standard deviation of 90 measured points; ^*c*^average chemical composition of basaltic glasses of ICDP HSDP2 drill core; ^*d*^standard deviation of 14 measured glasses.*

### Growth Medium

One of our main objectives was to investigate the release microbially induced release of Fe and other major elements from core samples to solution. The growth medium should therefore entirely exclude such elements or contain them in very low concentrations to facilitate their detection when released in low concentrations. We used a growth medium first described in Wu et al. (2007) for the experiments which contained only trace amounts of K, Mg, Mn, and Na and no Fe, Si, Al, Ca, and P (in the following denoted as M_-P_). This medium was used for incubation and colonization experiments. The major constituents were: glucose (0.2 g/L), NH_4_Cl (0.04 g/L), KCl (0.0005 g/L), and MgSO_4_ (0.0005 g/L). The medium was prepared with ultrapure H_2_O, the pH adjusted to 7 using ammonium hydroxide and sterilized by autoclaving at 121°C. Glucose was 0.2 μm sterile filtered and added after autoclaving. Furthermore, a second medium was prepared with the addition of NaH_2_PO_4_ and KH_2_PO_4_ 0.3 g/L each (in the following denoted as M_+P_). This medium was only used for preconditioning of the culture.

### Microorganism and Culture Preparation

We used the rod-shaped Gram-negative bacterium *B. fungorum* (ATCC BAA-463) first described in Coenye et al. (2001). It was found in basaltic aquifers of Snake River Plain as well as Hawaiian volcanic deposits (Sebat et al., 2003; Dunfield and King, 2005) and is known to weather rocks for nutrient acquisition (Wu et al., 2007; Mailloux et al., 2009). The microorganism was obtained from the German Collection of Microorganisms and Cell Cultures (DSMZ) in Braunschweig, Germany. A microbial strain of *B. fungorum* was grown for 24 h in tryptic soy broth complete medium (17 g/L casein peptone, 2.5 g/L K_2_HPO_4_, 2.5 g/L glucose, 5 g/L NaCl, 3 g/L soya peptone; Sigma-Aldrich) at 30°C on a shaking table. Five milliliter were removed and centrifuged at 5000 rpm for 10 min. The supernatant was discarded, the culture washed with 5 ml M_+P_ and centrifuged. This step was repeated three times. The washed cells were again cultured for 24 h in 50 ml M_+P_ at 30°C on a shaking table. A cell density of 10^8^ cells/ml was determined by counting with a hemocytometer. Five milliliter were removed, washed with M_-P_ and centrifuged. This step was repeated three times. A total of 50 μl of this culture were added to each assay in order to obtain an initial cell density of 10^5^ cells/ml.

### Incubation Experiments

Incubation experiments were performed to continuously monitor changes in solution chemistry between biotic and abiotic alteration of natural basaltic rocks from the HSDP2 drill core samples. The sample material was put in acid washed (5 M HNO_3_) 100 ml glass Erlenmeyer culture flasks and sterilized in an autoclave at 105°C by a three times repeated sterilization with cooling to room temperature in between in order to kill possible spores (tyndallization). Subsequently, the sample material was washed with sterile, ultrapure H_2_O to remove dissolved elements from possible mineral dissolution. The incubation experiments were conducted using 50 ml M_-P_ medium and a fluid:rock ratio of 100:1. Three biotic and three abiotic parallels (chemical control experiments) were prepared for incubation experiments for each assay. The culture flasks were incubated on a shaking table (125 rpm) at T = 8°C and 30°C for 41 days. The two different temperatures were chosen to simulate the large temperature differences in the borehole (see section “Site Description”). Sampling was done in time intervals (after days 1, 3, 9, 28, 41) and 6.5 ml aliquots were collected each time. 6.3 ml of these aliquots were passed through a 0.2-μm nylon syringe filter for element concentration and pH measurements, 100 μl were fixed in 2 % formaldehyde for total cell counting (SYBR green staining and fluorescence microscopy according to Hedrich et al., 2016) and 100 μl were used for glucose measurements.

### Colonization Experiments

Colonization experiments with synthetic basaltic glasses were performed to investigate the influence of Fe redox state and residual stress on microbial alteration and surface attachment of cells. A single melt droplet (about 1.5 g) of each sample was put in acid washed (5 M HNO_3_) 100 ml glass Erlenmeyer culture flasks and processed similarly than the natural samples (see section “Incubation Experiments”). Colonization experiments were conducted using 50 ml M_-P_ medium and a fluid:glass ratio of 50:1.5. For colonization experiments only two biotic parallels and one abiotic assay were prepared. The culture flasks were incubated on a shaking table (80 rpm) at *T* = 30°C for 42 days. No intermediate sampling was done for colonization experiments. After 42 days, 6.3 ml of the solution were collected and passed through a 0.2-μm nylon syringe filter for element concentration and pH measurements.

### Chemical Analysis

Glucose consumption and pH were measured immediately after sampling on filtered solutions with glucose test strips (MQuant glucose test) and a Knick pH-Meter 766 Calimatic electrode (uncertainty of <0.01 pH units), respectively. Glucose was measured to validate microbial growth in biotic experiments as well as absence of glucose consumption due to cell growth in abiotic controls. A total of 4.5 ml of the filtered solutions were acidified with 0.3 M HNO_3_ and stored at 4°C until elemental concentration analysis. Measurement of dissolved Si, Al, Fe, Mn, Mg, Ca, Na, K, and P was done by Inductively Coupled Plasma Optical Emission Spectroscopy (ICP-OES, Varian 725-ES).

Since fluid volume and element concentrations were decreasing with each sampling, the ICP-OES data were corrected as follows (Wu et al., 2007):

$$C_{j,i}^{*}=\frac{C_{j,i}[V_{0}-(j-1)V_{S}]+\Sigma_{h=1}^{j-1}C_{h,i}V_{S}}{V_{0}}$$

With $C_{j,i}^{*}$ being the corrected concentration of element *i* in the *j* sample, *C_j,i_* is the measured element concentration, *V_0_* is the initial fluid volume (0.05 L), *V_s_* is the sampling volume (0.0065 L) and $\Sigma_{h=1}^{j-1}C_{h,i}V_{S}$ accounts for the mass of element *i* extracted during sampling.

### Scanning Electron Microscopy (SEM)

The sample material from one of each parallels used in biotic incubation and colonization experiments was collected at the end of the experiments and critical point dried (Bray, 2000) for microscopic investigations of sample surface structures and biofilm formation. For this purpose, the cells were initially fixed with 3% glutaraldehyde in 10 mM HEPES buffer solution (pH 7) for 5 days at 4°C. Stepwise dehydration with graded series of 10, 30, 50, 70, 90, and ∼100 % ethanol was done for 10 min each. Following this, the samples were critical point dried to preserve cell morphologies, gold coated and stored in a desiccator until analysis. Samples were imaged on an Environmental Scanning Electron Microscope (SEM; FEI Quanta 200) equipped with an AMETEK New XL-30 EDX detector. A 20 kV accelerating voltage and a 10 mm working distance were used. All images were taken with a spot size 4 μm. Primary and secondary minerals as well as biofilm were identified using Energy Dispersive Spectroscopy (EDS) with a focused beam and a counting time of 100 s.

## Results

### Incubation Experiments

#### Glucose Consumption and Bacterial Growth

**FIGURE 3:**
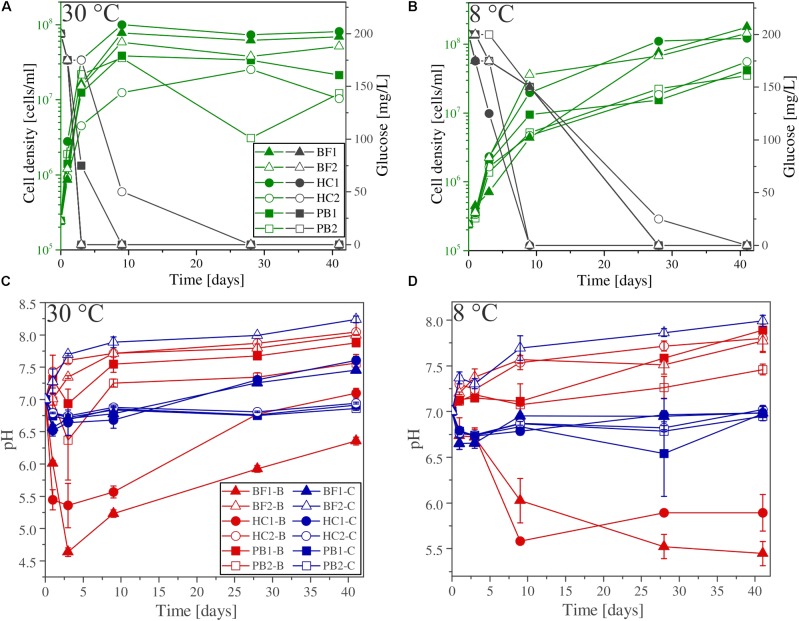
**(A,B)** Glucose consumption (black), cell growth (green), and **(C,D)** pH evolution for biotic (red) and abiotic (blue) incubation experiments versus time at 30°C and 8°C. Note that cell numbers were determined for one of the three parallels only. Error bars show the standard deviation of three parallel experiments. The sample suffixes –B and –C denote biotic and abiotic (control) experiments, respectively.

Glucose (200 mg/L) was added to all experimental assays (biotic and abiotic) as carbon source for microbial growth. The glucose consumption and cell densities were measured throughout the experiments in different time intervals. **Figures 3A,B** show the glucose consumption and cell densities of *B. fungorum* grown on natural basaltic rocks at 30°C and 8°C. Bacterial growth was evidenced in all inoculated experiments by glucose consumption. Within the first 3 days glucose was no longer measurable in all 30°C experiments except for sample HC2 for which glucose was completely consumed after 28 days. The same observations were made for the 8°C experiments but more than 4–20 days delay were observed. In abiotic controls for both, 30 and 8°C, no glucose consumption was observed and concentrations remained constant at the initial concentration. At the time glucose concentrations decreased in biotic experiments, cell densities increased from initially 10^5^ cells/ml to a maximum of 2⋅10^8^ cells/ml (BF1 and HC1) and reached a stationary phase after 9 days in 30°C experiments. Between days 9 and 41 cell densities remained constant at ∼10^8^ cells/ml. For samples BF2, HC2, PB1, and PB2 the same growth patterns were observed but with lower cell densities. In experiments at 8°C, no stationary phase was reached and cell densities increased till day 41 to more than 4⋅10^8^ cells/ml. The bacteria were uniform in shape (rod-shaped) and size (1–2 μm) throughout all biotic experiments as evidenced by fluorescence microscopy. Furthermore, no cells were detected in abiotic control experiments. We therefore conclude that abiotic controls remained sterile during the experiments.

#### Shift in pH

The shift in pH of the experimental solutions for abiotic and biotic experiments is shown in **Figures 3C,D**. For all abiotic controls pH slightly decreased during the first day by 0.5 log units and slowly approached to the initial pH 7 with time. An exception is sample BF2, where the pH increased to above 8. Considering biotic experiments at 30°C the pH for two of the samples strongly decreased in the first 3 days to 5.5 (HC1) and 4.5 (BF1) and increased with time to approach the initial pH 7. For sample HC2 and BF2 the pH rapidly increased to 7.5 and remained constant over time. For both pillow basalts (PB1 and PB2), the pH slightly decreased after day 1 followed by an increase to pH 7.5 and remained constant over time. For 8°C experiments, similar trends were observed but delayed by several days (e.g., the pH for samples BF1 and HC1 remained acidic until day 41).

#### Element Release

**FIGURE 4:**
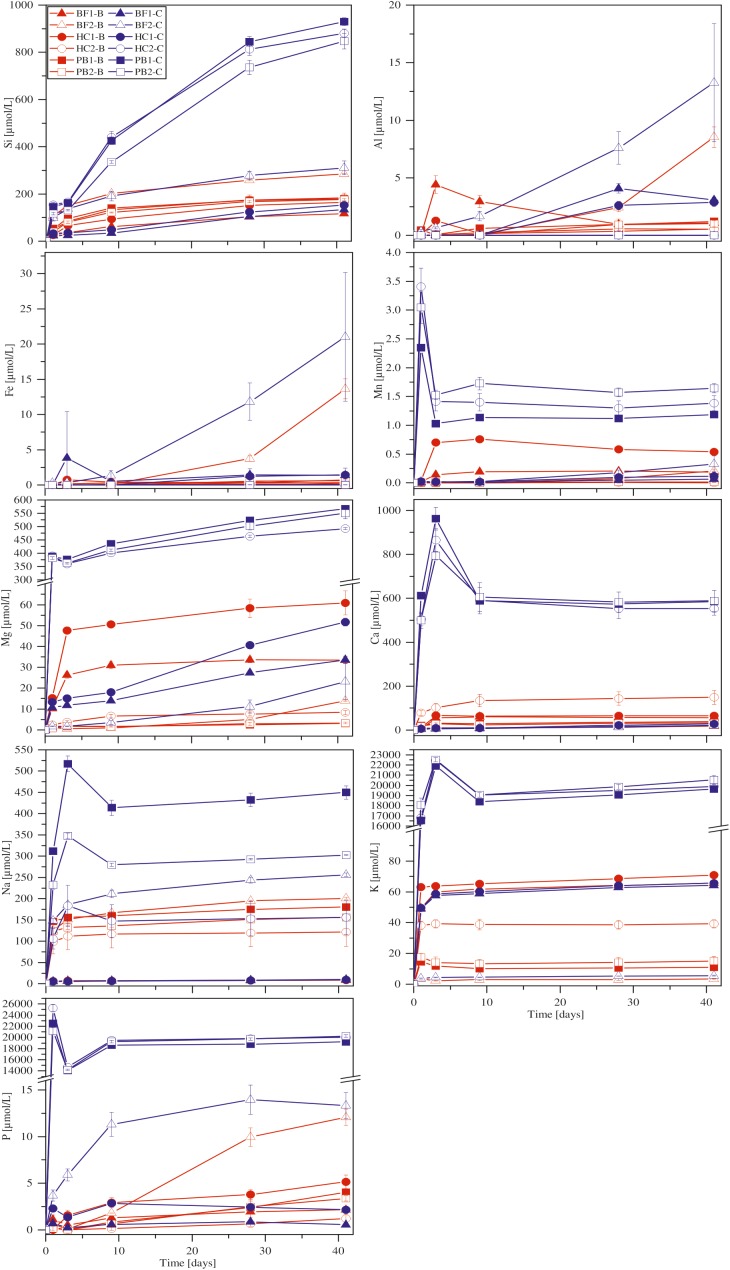
Concentration versus time of dissolved major elements in biotic (red) and abiotic (blue) incubation experiments at 30°C. Error bars show the standard deviation of three parallel experiments. The sample suffixes –B and –C denote biotic and abiotic (control) experiments, respectively.

With the exception of Ca, all other elements were initially below the detection limit in the starting medium (M_-P_). Evaporation of the liquid during the experiments was found to be linear and in total approximately 5.5 ml over 41 days. **Figure 4** shows the corrected dissolved elemental concentrations vs. time for incubation experiments with natural basaltic rocks at 30°C. For samples HC2, PB1, and PB2, concentrations of dissolved Si, Mg, Ca, K, Na, P, and Mn were higher at day 1 in abiotic experiments compared to their biotic analogs. In biotic and abiotic experiments, Si and Mg concentrations steadily increased until day 41. Ca, K, Na, P, and Mn concentrations in biotic experiments increased until day 3 and reached steady state conditions. In contrast, Ca, K, Na, P and Mn concentrations in abiotic experiments sharply decreased after day 1 or 3 and reached steady-state conditions by day 9. The patterns for Ca, K, Na, P and Mn in abiotic experiments might simply reflect the precipitation of secondary phases (Wu et al., 2007). Only the concentrations of dissolved Fe and Al were higher in biotic experiments than abiotic controls for samples HC2, PB1, and PB2. A distinct different trend was observed for samples BF1 and HC1. Here, the concentrations of dissolved Si, Mg, Ca, K, P, and Mn were lower in abiotic experiments compared to biotic analogs. The differences between biotic and abiotic experiments are not as pronounced as for the samples HC2, PB1, and PB2 with concentrations not more than a factor of 2 higher in biotic experiments. The concentrations of dissolved Fe and Al were higher in abiotic experiments. Dissolved Na was not measurable for samples BF1 and HC1. The concentrations of dissolved elements for sample BF2 were between those two groups (HC2, PB1, PB2 vs. HC1, BF1) described above with mostly higher concentrations in abiotic controls but nearly no dissolved K and Ca for biotic and abiotic experiments. For 8°C experiments the same trends were observed but with lower total element concentrations (for details see Supplementary Figure S1).

#### Surface Morphologies

**FIGURE 5:**
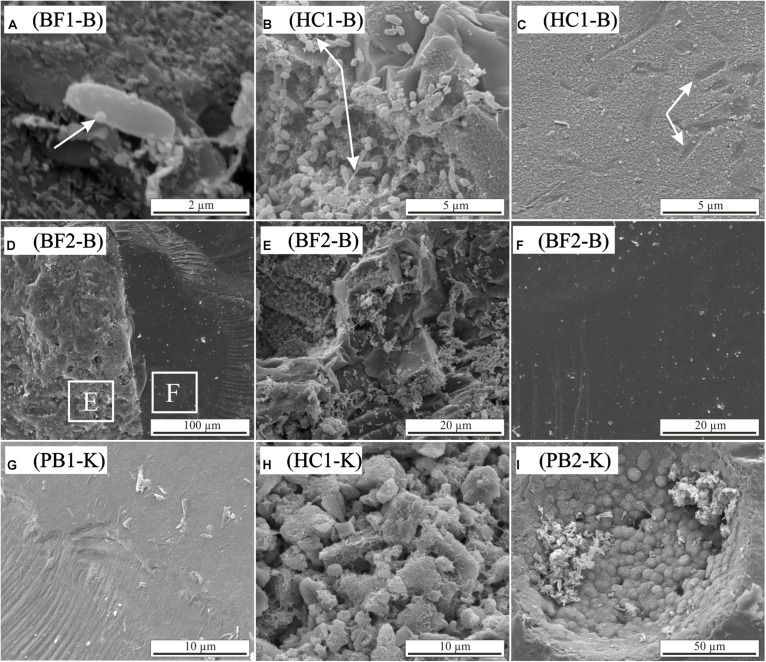
Scanning electron micrographs of biotic and abiotic incubation experiments with HSDP2 core samples (**A–F**: biotic experiments; **G–I**: abiotic experiments). **(A)** Single cell with small particles adhering to its surface. **(B)** Clusters of cells connected by filamentous structures. **(C)** Altered glass surface with possible imprints of microbial cells. **(D)** Basaltic flow (BF2) showing basaltic glass (left) and olivine crystal (right). **(E,F)** Enlargement of the areas indicated in image **(D)**. Glass **(E)** is intensively colonized by cells of *B. fungorum* whereas cells are absent on olivine surfaces **(F)**. **(G)** Abiotic altered glass surface covered with alteration products. **(H)** Small particles cemented in mesh-like alteration products. **(I)** Porous spherical structures in a primary vesicle.

To further investigate the attachment of microbial cells and precipitation of secondary minerals on surfaces during alteration of basaltic rocks and glasses the experimentally altered samples were analyzed using SEM. The following results are based on observations only and are not further quantified by statistical methods. Microbial cells of *B. fungorum* where found on all biotic incubated basaltic rocks in different quantities. **Figures 5A–F** shows secondary electron images of the biotic altered natural basaltic rock samples. Attached cells of *B. fungorum* appeared in rods with 0.5–2 μm length and small nm-sized particles often adhere to the cells (**Figure 5A**; arrow). Filamentous structures connecting individual cells were common when cells were located close to each other (**Figure 5B**). Compositional analysis of the filaments was not possible due to their small size (<0.1 μm). Glassy surfaces were in some places strongly altered and covered with precipitates. Such regions showed imprints that were well fitting in size to microbial rod-shaped cells (**Figure 5C**; arrows). **Figure 5D** shows basaltic glass (left site) and an olivine phenocryst (right site) in direct contact. The glass surface (**Figure 5E**) was intensively covered with cells of *B. fungorum* whereas the mineral surface (**Figure 5F**) was (almost) free of cells. Although the distance on the sample between **Figures 5E,F** was less than 100 μm, direct attachment of cells on smooth mineral surfaces was scarce and cells were often scattered. Cells appeared to be more abundant on glassy surfaces and particularly along fractures and holes. Apart from attached microbial cells, we did not observe major differences in surface morphologies between biotic and abiotic altered basaltic rocks. In **Figures 5G–I** abiotic altered samples are shown featuring characteristics that were observed for biotic as well as abiotic altered samples. Glass surfaces were in some parts intensely altered (**Figure 5G**) and in other parts without any signs of alteration (not shown here). Such fresh glass surfaces are probably fractured surfaces and a relic of sample preparation. Fine rock material accumulated along fractures was often covered and cemented with a mesh-like structure of secondary minerals (**Figure 5H**). In some cases, we observed porous spherical structures located in former primary vesicles of the basaltic glass (**Figure 5I**) and round spheres adhering to the surface (not shown). Qualitative EDX analysis indicated that the porous and round spheres were most probably zeolites and carbonates, respectively.

#### Colonization Experiments

The colonization experiments were primarily designed only to investigate the influence of the Fe redox state and residual stress on the activity and the interaction between microorganisms and basaltic glass. For that reason, pH and concentration of dissolved elements was measured at the beginning and upon termination of the experiments.

#### Shift in pH

It has to be noted that we do not have information about pH variations during the experiment and, thus, our observations refer only to the start and end of the experiment. The pH of the experimental solutions in abiotic controls did not show any systematic variations with changes in residual stress or Fe(II) content and decreased from initially pH 7 to ∼6.5 after 42 days (for samples BAS 1-C, BAS 2-C, BAS 3-C). Considering biotic experiments, the pH of the solution significantly decreased with increasing Fe(III) content from initially 7 to pH 6 (BAS 3-B) to pH 3.5 (BAS 1-B, BAS 2-B). We did not observe any effects of residual stress on the evolution of the pH (see also Supplementary Figure S2).

#### Element Release

**FIGURE 6:**
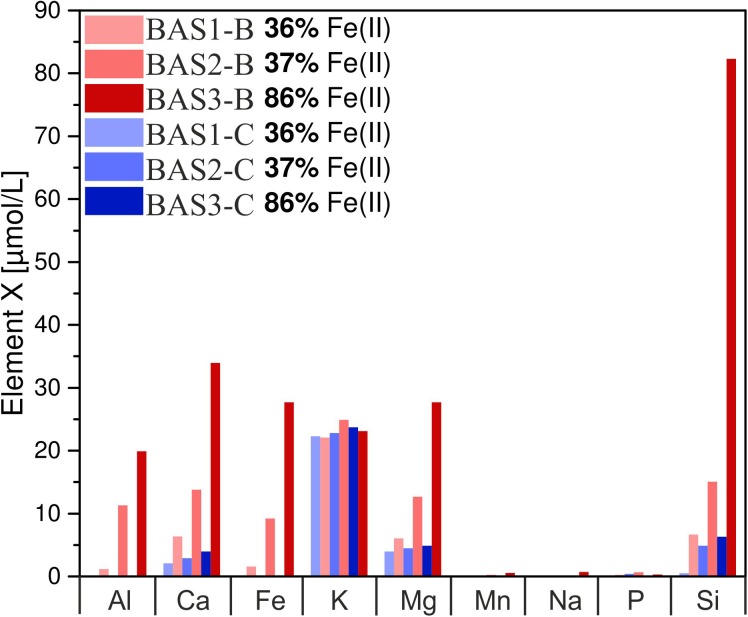
Concentration of dissolved major elements in biotic (red) and abiotic (blue) colonization experiments after 42 days. Note that concentrations have been measured in one of the parallels only and these data represent absolute values. The sample suffix –B and –C denotes biotic and abiotic (control) experiments, respectively. BAS1, annealed (no residual stress), low Fe(II) content; BAS2, quenched (residual stress), low Fe(II) content; BAS3, quenched (residual stress), high Fe(II) content.

The concentration of dissolved elements measured after 42 days is shown in **Figure 6**. In abiotic experiments, concentrations of dissolved Ca and Mg only slightly increased with residual stress and Fe(II) content in the glass whereas for Si differences were more pronounced. Dissolved Fe and Al were below the detection limit in all abiotic experiments. K concentrations were similar for all solutions and independent of Fe redox state and residual stress. Na, Mn, and P concentrations were close to the detection limit of the ICP OES in abiotic as well as biotic experiments and, hence, not part of further investigations. In biotic colonization experiments, concentrations of dissolved Si, Mg, Ca, Fe and Al increased with residual stress as well as with increasing Fe(II) content in the glass. Dissolved Si concentrations increased from 7 to 15 μmol/L (BAS1 → BAS2) and further to 82 μmol/L (BAS2 → BAS3) with increasing Fe(II) content in the glass. Ca and Mg concentrations increased by a factor of 2 with increasing residual stress in the glasses (BAS1 → BAS2) and again by more than a factor of 2 with increasing Fe(II) content (BAS2 → BAS3). Dissolved Fe and Al were detected in biotic experiments only, and their concentrations strongly depended on both, residual stress (increase from 1.5 to 9 μmol/L) and Fe redox state (further increase from 9 to 28 μmol/L). K concentrations were found to be independent from residual stress and Fe(II) similar to abiotic experiments.

#### Surface Morphologies

**FIGURE 7:**
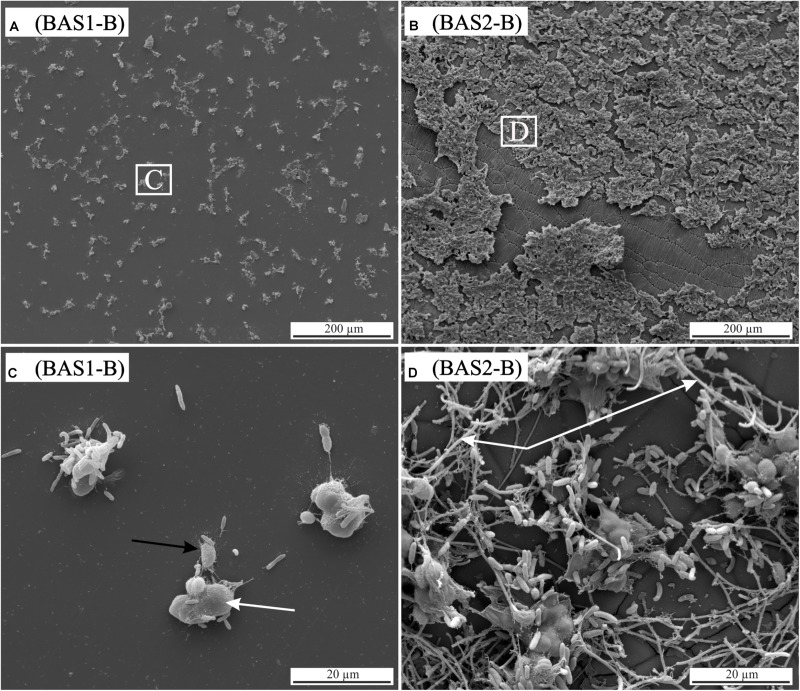
Scanning electron micrographs of biotic colonization experiments with synthetic basaltic glasses. **(A)** BAS1 [no residual stress; low Fe(II) content] with clustered cells scattered on the surface. **(B)** BAS2 [residual stress; low Fe(II) content] showing intense colonization and biofilm formation on the surface. Note also the network of cracks that became altered during the experiment. **(C)** Enlargement of the area indicated in image **(A)**. Two cell types were observed: cells comparable in shape and size to those observed in incubation experiments and large (up to 10 μm), round shaped cells forming colonies (white arrow). Filamentous structures (nm sized) seem to facilitate cell-surface attachment (black arrow). **(D)** Enlargement of the area indicated in image **(B)**. A second type of filamentous structures (μm sized) was observed connecting the colonies (white arrow).

**FIGURE 8:**
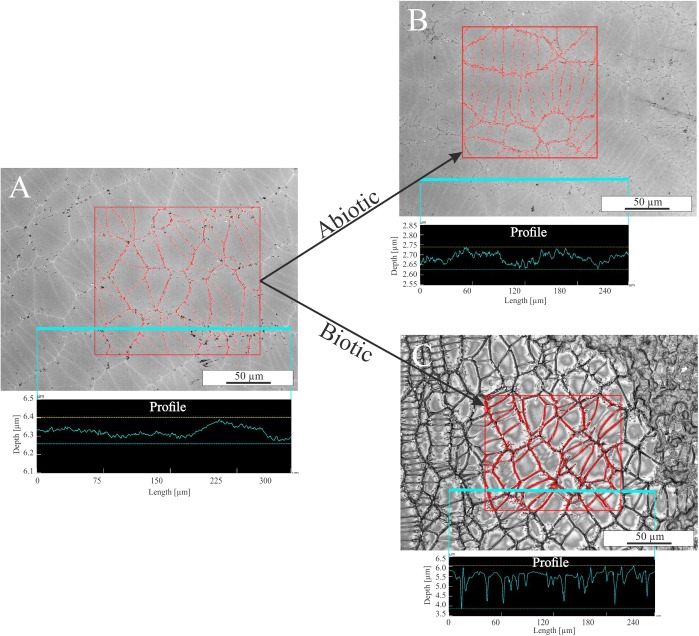
Laser scanning microscopy images of glass BAS3 surfaces before and after biotic and abiotic experiments. **(A)** Glass surface prior to the experiments. A fine network of stress-induced cracks is visible. **(B)** Glass surface after abiotic colonization experiment. Some of the cracks are slightly altered but changes are negligible. **(C)** Glass surface after biotic colonization experiments. The entirety of the cracks is intensively altered and the surface is covered with biofilm (upper right corner). The measured profiles indicate that cracks are not deepened prior to and after abiotic experiments [see **(A,B)**] but are up to 2 μm deep after biotic alteration [see **(C)**]. Note the different scales on the *y*-axis for the profiles.

The influence of single parameters such as Fe redox state and residual stress on microbial alteration as well as the attachment of cells on surfaces were investigated in detail in colonization experiments with synthetic basaltic glasses. The following results are based on observations only and were not further quantified by statistical methods. Nevertheless, the images are representative for the entire sample and no strong variations were observed (see Supplementary Figure S3). Compared to the incubation experiments with natural samples, cells adhering to the surface and biofilm formation on it were more pronounced (**Figure 7**). The abundance of cells and the extent of the biofilm on the glass surface seemed to depend on the residual stress and only to a minor extent on the Fe(II) content of the glass. Considering the residual stress, glass BAS1 (annealed = no residual stress) was sparsely colonized with cells and biofilm that were scattered over the glass surface (**Figure 7A**). With increasing residual stress the abundance of cells and the extent of biofilm formation seemed to increase for sample BAS2 (**Figure 7B**). The morphology of the cells was not as uniform as observed in incubation experiments and could be distinguished by two different forms: Cells similar in shape and size to those described previously and cells encapsulated in biofilm. Those encapsulated cells were round shaped, up to 10 μm in diameter and grouped together to form colonies (**Figure 7C**; white arrow). With the presence of two types of cells, we also observed two types of filamentous structures. The first was comparable to those observed in incubation experiments. The filaments were a few nm thin, associated with individual cells and seemed to facilitate direct attachment of cells to the glass surface (**Figure 7C**; black arrow). The second type of filaments was much thicker (up to 1 μm) and connected the previously described colonies (**Figure 7D**; arrow). The biofilm did not entirely cover the glass and seemed to be partly disrupted. We assume that this had happened during sample preparation for SEM since the critical point drying process involves several steps that could damage the biofilm. Comparison of the unaltered and altered glass surfaces by laser scanning microscopy showed considerable differences in surface alteration between biotic and abiotic experiments (**Figure 8**). The quenched glasses (BAS2 and BAS3) exhibited a fine network of cracks at the surface related to thermal contraction at the interface between the melt and air during cooling (**Figure 8A**). Analysis of images from laser scanning microscopy showed that such cracks were closed before the experiments (**Figure 8A**; profile). In abiotic experiments, the cracks were partially altered and deepened after 42 days (**Figure 8B**; profile). The surface did not show any signs of dissolution or mineral precipitation. In biotic experiments, however, alteration of the surface was much stronger pronounced. All cracks were intensively altered and deepened up to 2 μm (**Figure 8C**; profile). The glass surface had become rough, possibly by precipitation of secondary phases. We did not observe signs of individual etch pits. The intensity of the glass surface alteration was found to be mainly related to residual stress and only to a minor extent to the Fe(II) content.

## Discussion

### Microbial Growth With Basaltic Rocks as a Nutrient Source

Microbial life is ubiquitous in the oceanic crust and contributes a substantial part to geochemical element cycling (Staudigel et al., 1995; Thorseth et al., 1995b; Furnes and Staudigel, 1999; Bach and Edwards, 2003). It is well known that microorganisms are capable of producing organic acids and metal specific organic ligands to sequester essential nutrients (Kraemer, 2004; Rogers and Bennett, 2004; Perez et al., 2016). Some metal-oxidizing bacteria are also able to biological catalyze Fe(II) or Mn(II) oxidation for energy gain (Templeton et al., 2005; Bailey et al., 2009; Emerson et al., 2010; Henri et al., 2016; Sudek et al., 2017). However, there is still uncertainty about whether microorganisms actively scavenge elements from the rock or simply incorporate elements that are released by abiotic weathering processes (Thorseth et al., 1992, 1995a,b; Templeton et al., 2009; Cockell et al., 2010; Stockmann et al., 2012; Ward et al., 2013).

In our incubation experiments with natural basaltic rocks we observed a marked uptake of elements by *B. fungorum* from solution. Considering **Figure 4** the uptake of elements from solution by *B. fungorum* in biotic incubation experiments was high for samples HC2, PB1, and PB2, e.g., concentration of dissolved Ca in abiotic controls was >500 μmol/L compared to <200 μmol/L in biotic experiments. These samples were characterized by high SSAs (>30 m^2^/g). Deviations from the aforementioned behavior included samples BF1 and HC1 with low SSAs of 3.05 and 3.07 m^2^/g, respectively. Here, the concentration of released elements was lower in abiotic controls compared to the biotic experiments, e.g., concentration of dissolved Ca in chemical controls was <30 μmol/L compared to >50 μmol/L in biotic experiments. These distinct trends were observed for all major elements except Fe and Al. Concentrations of dissolved Fe were low for most samples in both, biotic and abiotic experiments. However, only from these observations it cannot be deduced that basaltic rocks from HSDP2 drill core are a nutrient source and facilitate growth of *B. fungorum*. As a consequence of autoclaving at 105°C large amounts of elements were released into solution possibly by dissolution of secondary minerals. Removal of such elements by washing with sterile H_2_O was not sufficient enough and relics of secondary minerals may be rapidly dissolved at the beginning of the experiments resulting in high initial concentrations of Mn, Mg, Ca, Na, K, and P in the solution (see **Figure 4**). If we consider a marked release of elements during autoclaving, one would expect such elements being released in all assays (biotic and abiotic) in about the same quantity. Although a portion of the elements released during autoclaving in biotic assays were most probably scavenged by *B. fungorum* we do not have clear indication that the bacteria actively participated in dissolution of the basaltic rocks in incubation experiments.

By contrast, the results of colonization experiments with synthetic basaltic glasses clearly showed that *B. fungorum* was capable of obtaining nutrients directly from the glass instead of passively scavenging dissolved elements as stated above. First, the release of major elements during sterilization (autoclaving) of synthetic basaltic glasses was lower compared to the natural basaltic rocks of HSDP2 drill core. Hence, the environment in colonization experiments was supposed to be nutrient depleted in the initial stages of the experiments. Second, the presence of *B. fungorum* strongly enhanced the basaltic glass dissolution and the release of major elements to solution relative to abiotic controls (see **Figure 6**). This is most pronounced for dissolved Fe and Al which were detected in biotic experiments only. The basaltic glass in colonization experiments was the only source for the microbes to obtain essential nutrients such as Fe and P. It is therefore most likely that *B. fungorum* was forced to actively scavenge nutrients for microbial growth and metabolism in colonization experiments from the basaltic glasses (in contrast to a nutrient rich environment in incubation experiments).

The low concentrations of dissolved Fe in biotic incubation experiments are in contrast to what was observed in biotic colonization experiments with synthetic basaltic glasses. Due to the relatively high concentrations of dissolved P in incubation experiments (up to 18 mmol/L for samples HC2, PB1, and PB2) it is possible that the Fe had been precipitated as Fe-phosphate. However, we did not observe any Fe-phosphate precipitates at the surface of the natural samples during SEM analysis. Wu et al. (2007) also investigated the interaction of *B. fungorum* with basaltic rocks and found relatively high (up to 15 μmol/L) concentrations of Fe but no P in their biotic experiments. They assigned the release of Fe in the experiments to microbially produced chelators. Besides from experiments with a minimal growth medium (M_-P_; see section “Growth Medium”) they also performed experiments with a P-rich medium (2.5 mmol/L P) and here, no dissolved Fe was detected in biotic experiments. Comparing the observations of Wu et al. (2007) and those of our study, it seems likely that P was a limiting factor for the growth of *B. fungorum*. In case the solution was depleted in P, the release of Fe from the glass/rock was higher than its uptake by the microbes and the concentration of Fe in the solution increased. If the solution was enriched in P, all the dissolved Fe was either used by *B. fungorum* or precipitated as secondary Fe-phosphates. This would mean that P was preferentially dissolved from the glass which also contained Fe in appreciable amounts. This assumption is also supported by colonization experiments. Here the concentration of dissolved P in biotic experiments was close to the detection limit for all glasses (see **Figure 6**). Dissolved Fe, on the other hand, was detected in all biotic experiments in appreciable amounts. The need of some microorganisms for P was also evidenced by Bailey et al. (2009) in laboratory experiments. Microbial growth on basaltic glasses was enhanced on those doped with apatite as P source. From our observations it seems to be likely that in case *B. fungorum* was not able to obtain nutrients from solution only, the organism started to attack the rock/glass possibly by lowering the pH (as observed for samples HC1/BF1 and BAS1/BAS2) to acquire essential nutrients and thus increasing the overall dissolution rate.

### Factors Influencing the Intensity of Microbial Alteration

#### Specific Surface Area

The SSA is an important parameter as it determines the reactive surface susceptible to chemical exchange with the environment and might also have an influence on microbial behavior during alteration. A high release of Si, Mn, Mg, Ca, Na, K, and P to solution for samples with high SSA was observed. As previously mentioned we ascribe this release to be an artifact resulting from autoclaving. For instance, the results from the sequential extractions (see section “Preparation of Core Samples”) provide evidence that for samples with a high SSA (BF2 = 28.89 m^2^/g, HC2 = 33.73 m^2^/g, PB1 = 20.23 m^2^/g, PB2 = 43.78 m^2^/g) the amount of amorphous secondary Fe, Al, and Si phases are five times higher (Ø 484 μmol/g) compared to samples with a low SSA (BF1 = 3.05 m^2^/g, HC1 = 3.07 m^2^/g) (Ø 85 μmol/g). Dissolution of these amorphous secondary phases during autoclaving is favored as an explanation for unusual high initial dissolved element concentrations. It is therefore difficult to ascribe specific observation in our experiments to the SSA. Nielsen and Fisk (2010) measured SSA’s of different extrusive volcanic rocks of the oceanic crust and inferred that the SSA is not a limiting parameter for biomass production and cell attachment. From our observation it seems likely that SSA contribute only to a minor part to the microbial alteration rate and is a parameter mainly controlling elemental release rates during abiotic alteration.

#### Fe Redox State

The Fe redox state of basaltic rocks from the ICDP drill core is a mixture of different Fe sources from primary minerals [e.g., Fe(II) from olivine], glass [Fe(II) and Fe(III)] and secondary minerals [mainly Fe(III)]. Thus, the Fe redox state was quite heterogeneous and varied in the same sample making predictions about its contribution to microbial alteration difficult. For this reason, chemically homogeneous synthetic basaltic glasses with well-known Fe redox state were used as an analog for natural glasses. We observed that the concentration of dissolved Fe and other major elements in biotic colonization experiments increased with increasing Fe(II) content (see **Figure 6**). In abiotic colonization experiments the concentration of dissolved elements (except Fe and Al) increased with increasing Fe(II) content as well but less pronounced compared to biotic experiments. Within this study, it was not our goal to point out the mechanisms by which *B. fungorum* participated in the basalt dissolution. However, in the following we will try to discuss possible mechanisms that seem reasonable to us.

A variety of marine Fe oxidizing microorganisms (FeOB) such as the neutrophilic, chemolithotrophic bacterium *Mariprofundus ferrooxydans* are able to grow on basaltic glasses and are capable of using Fe(II) as an energy source (Henri et al., 2016). However, abiotic oxidation and hydration of Fe(II) at circumneutral pH and oxygenated conditions are fast (Davison and Seed, 1983). Microorganisms circumvent this problem by living in an acid or microaerophilic environment where Fe(II) is more stable (Emerson et al., 2010). Both of the aforementioned situations are not applicable to our experiments. Changes in the solution pH were not restricted to the silicate-biofilm interface (Liermann et al., 2000) but affected the whole solution. Moreover, pH variations were not uniform among the samples and for some the pH became basic with time, thus, it seems unlikely that *B. fungorum* used Fe(II) as an energy source.

The chemical durability of silicate glasses is mostly controlled by their composition. Network formers such as Si and Al increase the chemical durability by polymerizing the glass structure whereas network modifiers such as Na and Ca weaken the glass structure (Scholze, 1991). Fe(III) has been shown to act as a network former similar to Al(III) increasing the chemical durability of glasses (Paul and Zaman, 1978). Fe(II) on the other hand was found to be a network modifier depolymerizing the glass network and decreasing its chemical durability (Kumar, 1991). The pH changes observed in colonization experiments for high Fe(III) glasses (BAS1/BAS2) can therefore be considered as a reaction of *B. fungorum* to release nutrients from the chemically more durable glass possibly by production of organic acids. This assumption is also promoted by observations from incubation experiments.

As mentioned before, crystalline basaltic rocks are chemically more resistant than basaltic glasses. The solution in experiments with crystalline samples and low SSA (BF1/HC1) became acidified whereas an increase to slightly basic pH was observed for those rocks with high amounts of glass and high SSA (see **Figure 3**). Finally, microbial cells were absent on olivine crystals containing solely Fe(II) but abundant on glassy parts with at least 10 % of its total Fe being Fe(III). Considering **Figure 6**, dissolved Fe and Al were only measured in biotic colonization experiments. Moreover, the concentrations of dissolved Fe and Al increased in the same manner. We have no knowledge about the redox state of the dissolved Fe but based on our experimental conditions it is likely that all dissolved Fe was present as Fe(III). Perez et al. (2016) have shown that during interaction of *Pseudomonas aeruginosa* with basaltic glasses of various Fe redox states siderophore production increases with decreasing Fe(III) in the glass. In a previous study, trivalent metal chelators were found to interact with both, Fe(III) and Al(III), enhancing the overall glass dissolution (Perez et al., 2015). Simultaneous microbial dissolution of Fe(III) and Al(III) and an increased overall glass dissolution was also observed in colonization experiments during this study indicating the presence of microbial produced chelators. In another study *Pseudomonas stutzeri* VS-10 exhibited elevated growth in the presence of basaltic glass in Fe-limited heterotrophic media (Sudek et al., 2017). We do not know if *B. fungorum* is capable of producing such metal specific chelators even if some authors suggest it. An alternative hypothesis would be that the release of Fe and Al was induced by pH lowering. In silicate glasses, large amounts of Fe and Al can be extracted at acidic pH ≤ 3 (Paul and Zaman, 1978). However, in the range from pH 4 to 9, Fe and Al are stable and their dissolution rates are low. Considering our colonization experiments, the release of Fe and Al were highest at near neutral pH (glass BAS3; pH 6). There is the possibility that the pH dropped during the initial stage of this experiment similar to samples BAS1 and BAS2, and approached to near neutral conditions with time possibly by proton consumption. In this case we simply wouldn’t have sampled the solution at the time at which the pH was acidic.

#### Residual Stress

During our colonization experiments, basaltic glasses with residual stress were less chemically resistant than without under abiotic as well as biotic influences. However, whereas the concentration of dissolved elements in abiotic controls increased slightly with increasing residual stress, the effect was more pronounced in biotic experiments where concentrations were twice as high. Furthermore, cells of *B. fungorum* seemed to attach in greater numbers on glasses with residual stress compared to stress-free (annealed) glasses. We also observed a network of fine cracks beneath the surface of the quenched glasses (BAS2; BAS3), that was intensively altered in biotic experiments (see **Figure 8**). Dissolution was strongest along these weaker zones leaving the central parts relatively unaltered. It is quite clear that this alteration was solely caused by *B. fungorum*. Unfortunately, little is known about the effect of residual stress on microbial alteration so far. Dissolution of silicate minerals and glasses is not uniform and preferred in places with microfractures and dislocations (Stumm, 1992). Residual stress in quenched glasses favors fracturing which results in an increase in the SSA exposed to solution and, hence, increasing dissolution rates. Rosso et al. (2003) have shown that the bacterium *Shewanella putrefaciens* dissolves the surface of hematite crystals at locations that are energetically favorable and distinct from its point of attachment. Considering that volcanic islands as well as the oceanic crust hold large amounts of volcanic glass, residual stress can be a significant factor controlling the extent of alteration.

### Cell Attachment and Biofilm Formation

Biofilm formation is widespread among continental and oceanic volcanic rocks but significantly more distinct in aqueous, nutrient rich environments (Templeton et al., 2005; Herrera et al., 2008; Bagshaw et al., 2011; Callac et al., 2013; Sudek et al., 2017). Microorganisms associated with seafloor basalts can have various morphological appearances from individual or clustered cells to extensive biofilm formation adhering to surfaces (Einen et al., 2006; Templeton et al., 2009). Whether microbes attach to mineral surfaces depends on the availability of nutrients necessary for metabolic processes and microbial growth (Roberts, 2004; Rogers and Bennett, 2004; Bailey et al., 2009). The circumstances that promote surface attachment of microorganisms are diverse but it is ambiguous if direct attachment to mineral and glass surfaces is common or if it is a microbial reaction of changing environmental influences.

Our experimental findings indicate that cell attachment on surfaces is complex and influenced by several specific parameters. Observations from our incubation experiments with natural rock samples indicate that in a nutrient rich environment (as for samples HC2, PB1, and PB2) cells are occasionally found on surfaces. Under nutrient limited conditions (as for samples BF1 and HC1), attachment of microbial cells to glass surfaces appeared to be more pronounced. Based on nutritional conditions, McEldowney and Fletcher (1986) also reported a strong influence of the nutrient availability on the affinity of some freshwater microorganisms to attach on solid surfaces with greater attachment under nutrient-limiting conditions. Another interesting observation was the preference of *B. fungorum* for attachment on glass surfaces rather than mineral surfaces (e.g., olivine; see **Figures 5E,F**). We suppose that *B. fungorum* specifically attacked the basaltic glass to acquire P and Fe. This diea is in line with the results from our colonization experiments and findings of Rogers and Bennett (2004). Also, the fact that we did not observe apatite in our natural samples supports this assumption as basaltic glass is the only P source for *B. fungorum* in incubation experiments.

Indication that a nutrient depleted environment favors surface attachment was also obtained from colonization experiments. Furthermore, the abundance of attached cells seemed to be positively correlated with residual stress in the order BAS1 < BAS2/BAS3. The cell and biofilm morphologies in colonization experiments were more diverse than in incubation experiments. The nm sized filamentous structures seemed to facilitate either connection of cells among each other or to the glass surface. The larger filaments only observed in colonization experiments seem to connect solely the larger cells. The use of such structures linking individual cells is still not completely understood but assumed to play a role in electron transport between bacteria (Pirbadian et al., 2014; Sudek et al., 2017). The observations from incubation and colonization experiments of this study indicate that surface attachment of microorganisms might be favored in nutrient depleted environments and can possibly be an adapted strategy under extreme conditions.

## Conclusion

Understanding microbe–mineral interactions is of importance for geochemical element cycles and in particular the release of limiting nutrients (e.g., Fe and P) from rocks to the environment. We have shown that *B. fungorum* is capable of obtaining essential nutrients by itself via dissolution of basaltic glasses. Our study provides new insights about how rock properties, such as Fe redox state and residual stress, can affect microbial rock colonization and weathering. With increasing residual stress or high Fe(II) content, the synthetic basaltic glasses became more susceptible for microbial alteration. Furthermore, a correlation between nutrient-limiting conditions and surface attachment of microorganisms was emphasized. Cells of *B. fungorum* preferentially attached on glass surfaces of natural basaltic rocks that contained beneficial nutrients. It was also shown that biotic alteration could significantly contribute to the release of major elements in a nutrient depleted environment when microorganisms are forced to actively scavenge nutrients from rocks. The need of *B. fungorum* to acquire P for microbial growth was found to enhance the release of Fe and other elements from synthetic basaltic glasses to solution and is in agreement with previous studies (Wu et al., 2007). However, the amount of elements released in biotic experiments might be an underestimation and elements such as Fe and P, that are nutrients for microorganisms, were located and fixed (possibly unavailable for liquid phase analysis) in the biomass.

There are some limitations when transferring the findings of this work to the larger environment. The experiments were performed with a single bacterial species whereas natural rocks comprise diverse microbial communities that interact with each other. Furthermore, microbial growth was enhanced by adding glucose as a carbon source. The growth medium was prepared with a minimum concentration of salts (comparable to ground waters) to be able to measure even small amounts of elements released from the rocks and glasses. Our experiments are therefore not directly applicable to seawater conditions. Despite these limitations, the findings of this study are of great importance for the element release during weathering of volcanic islands and all leaching systems with highly permeable rocks. Within the highly porous rocks that are ubiquitous on the island of Hawaii, marked shares of soluble chemical weathering products are immediately transported away leaving a nutrient depleted environment behind that can favor microbial alteration.

## Author Contributions

AS, SD, and HB designed this study. MS conducted the laboratory work, analyzed the data, and wrote the manuscript. All authors discussed the data and revised the manuscript.

## Conflict of Interest Statement

The authors declare that the research was conducted in the absence of any commercial or financial relationships that could be construed as a potential conflict of interest.
