# Supplementary figures and images for: Experimental Microbial Alteration and Fe Mobilization From Basaltic Rocks of the ICDP HSDP2 Drill Core, Hilo, Hawaii
Source: Front Microbiol. 2018 Jun 14;9:1252. doi: 10.3389/fmicb.2018.01252 (PMC6010528; doi:10.3389/fmicb.2018.01252)

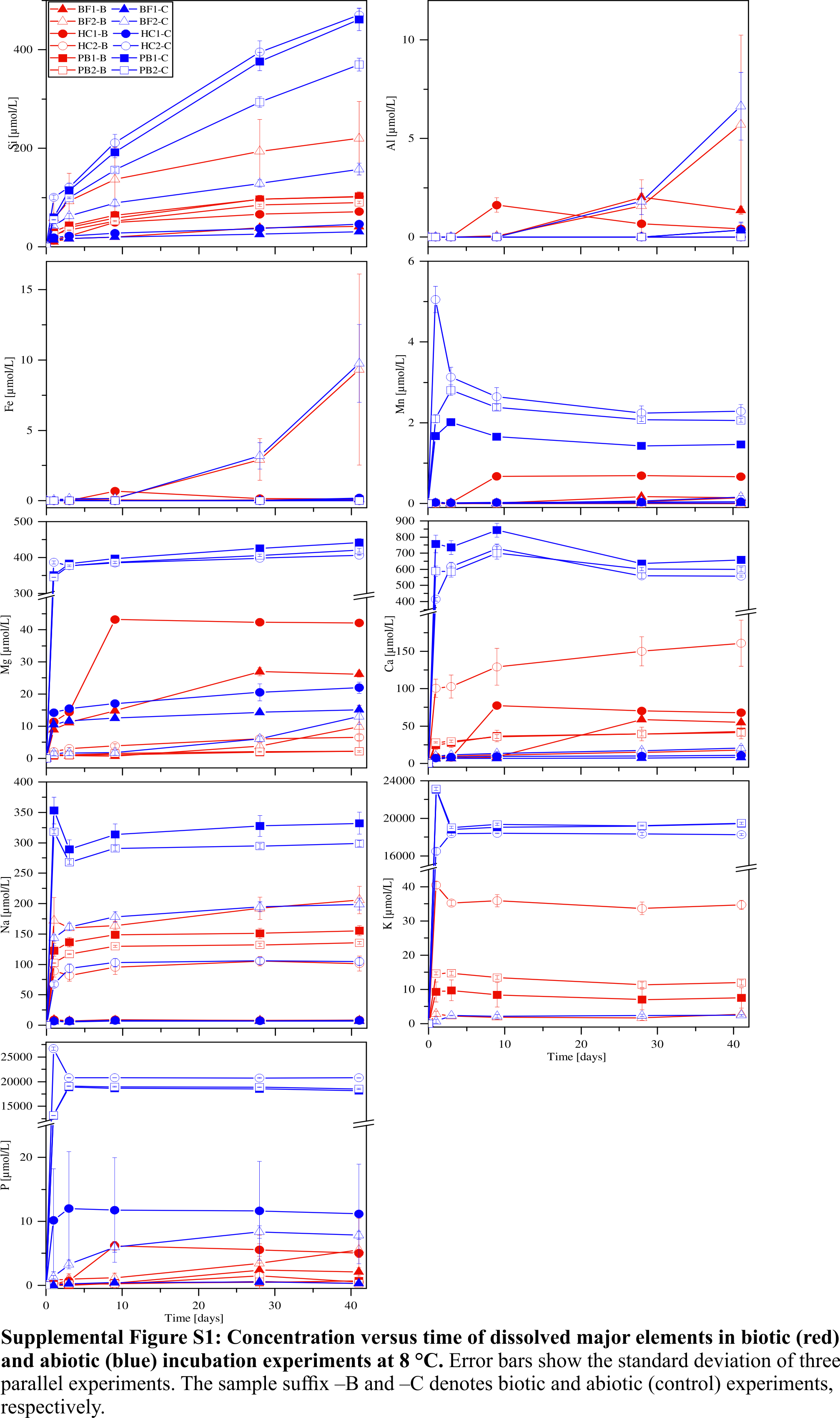

Supplement: Supplementary file 1 [file Image_1.jpg]

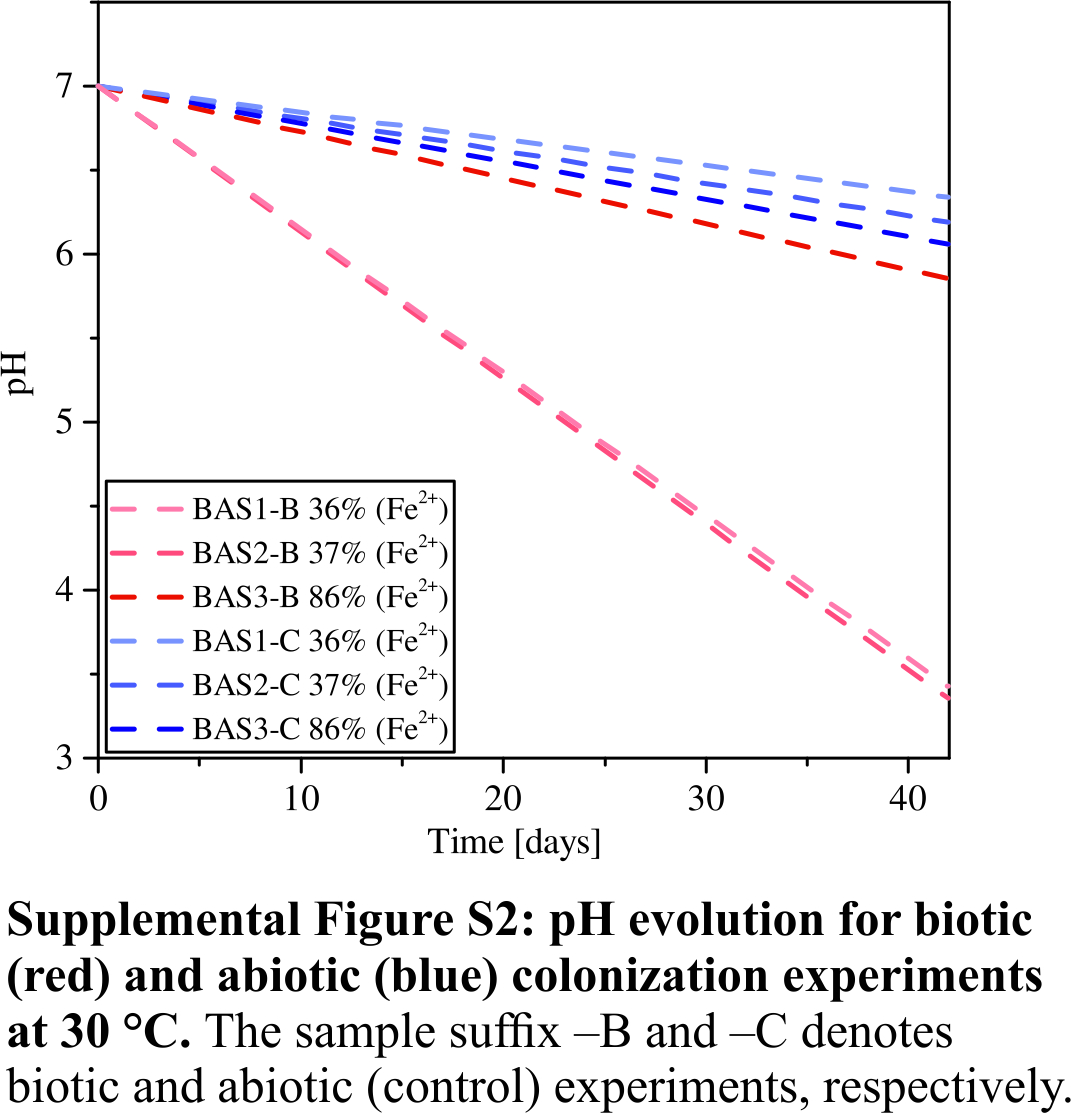

Supplement: Supplementary file 2 [file Image_2.jpg]

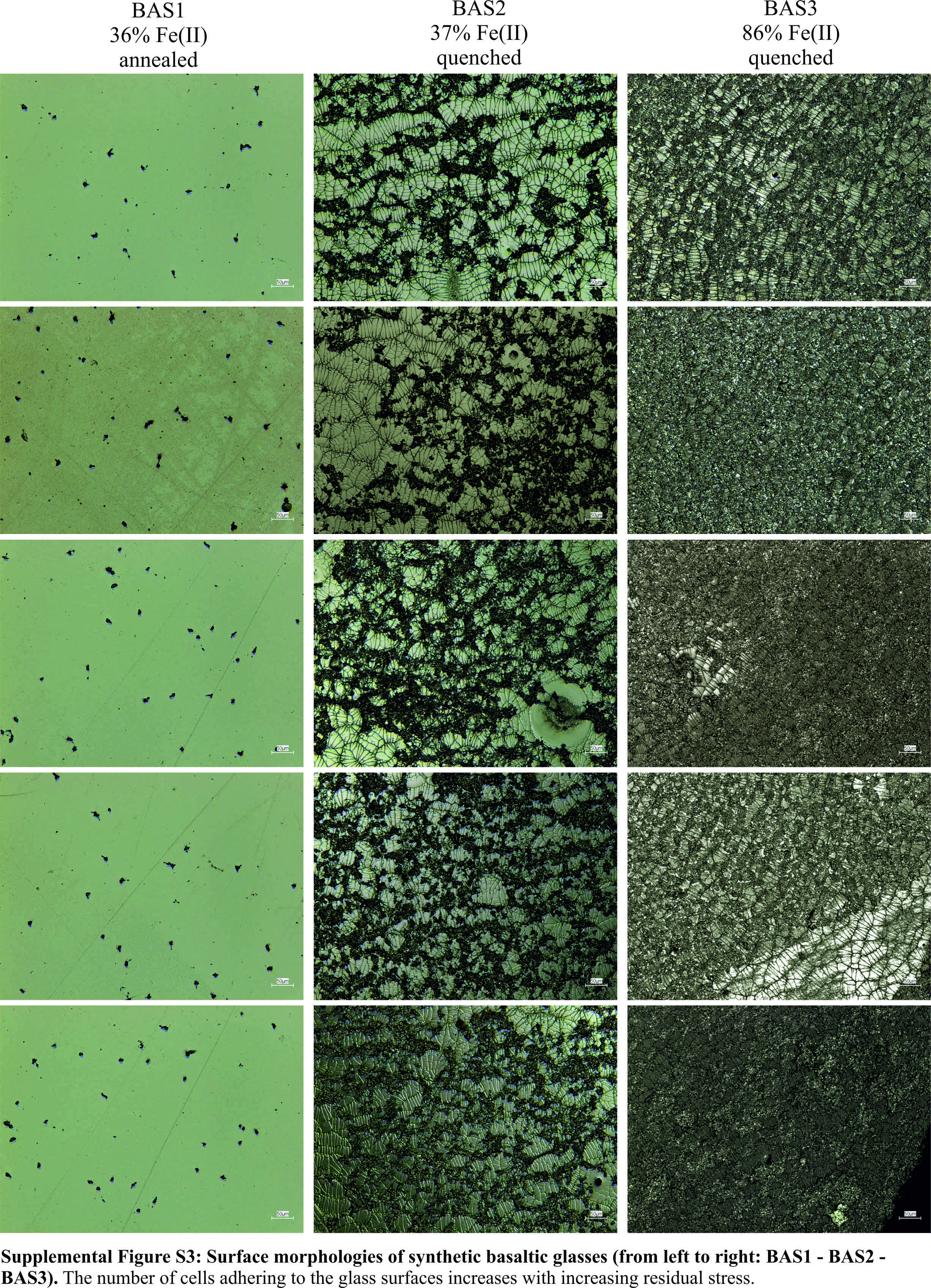

Supplement: Supplementary file 3 [file Image_3.jpg]
